# Enhanced Photoluminescence and Raman Properties of Al-Doped ZnO Nanostructures Prepared Using Thermal Chemical Vapor Deposition of Methanol Assisted with Heated Brass

**DOI:** 10.1371/journal.pone.0121756

**Published:** 2015-03-10

**Authors:** Tamil Many K. Thandavan, Siti Meriam Abdul Gani, Chiow San Wong, Roslan Md. Nor

**Affiliations:** 1 Plasma Technology Research Centre, Department of Physics, University of Malaya, Lembah Pantai, Kuala Lumpur, Malaysia; 2 Low Dimensional Material Research Centre, Department of Physics, University of Malaya, Lembah Pantai, Kuala Lumpur, Malaysia; Institute for Materials Science, GERMANY

## Abstract

Vapor phase transport (VPT) assisted by mixture of methanol and acetone via thermal evaporation of brass (CuZn) was used to prepare un-doped and Al-doped zinc oxide (ZnO) nanostructures (NSs). The structure and morphology were characterized by field emission scanning electron microscopy (FESEM) and x-ray diffraction (XRD). Photoluminescence (PL) properties of un-doped and Al-doped ZnO showed significant changes in the optical properties providing evidence for several types of defects such as zinc interstitials (Zn_i_), oxygen interstitials (O_i_), zinc vacancy (V_zn_), singly charged zinc vacancy (V_Zn_^-^), oxygen vacancy (V_o_), singly charged oxygen vacancy (V_o_^+^) and oxygen anti-site defects (O_Zn_) in the grown NSs. The Al-doped ZnO NSs have exhibited shifted PL peaks at near band edge (NBE) and red luminescence compared to the un-doped ZnO. The Raman scattering results provided evidence of Al doping into the ZnO NSs due to peak shift from 145 cm^-1^ to an anomalous peak at 138 cm^-1^. Presence of enhanced Raman signal at around 274 and 743 cm^-1^ further confirmed Al in ZnO NSs. The enhanced D and G band in all Al-doped ZnO NSs shows possible functionalization and doping process in ZnO NSs.

## Introduction

The native nature of zinc oxide (ZnO) as an intrinsic n-type semiconductor has embarked several favorable properties, including good transparency, high electron mobility 2000 cm^2^/(Vs) at 80 K [1], wide band gap 3.37 eV and strong room temperature luminescence [2]. The wide band gap which lies in the ultraviolet (UV) region and alternating stacking layers of Zn and O has made ZnO a unique candidate for UV light lasers and detectors working in the range of 368–390 nm [3,4]. In addition to these properties, ZnO emits different color lights and all constituents of white light due to large number of extrinsic and intrinsic deep-level impurities and clusters [5–7]. These properties have created applications for various fields; transparent electrodes in liquid crystal displays (LCD), energy saving and heat protecting windows as well as in the field of electronics as thin film transistors and light emitting diodes (LED). Thus, many investigations and researches based on growth techniques have been employed and reported elsewhere achieving un-doped and doped ZnO in one-dimensional (1D) nanostructures (NSs) such as nanotubes [8], nanowires [9], nanorods [10,11], nanobelts [12], nanocables [13], and nanoribbons [14]. Elements like Al, In, Ga, etc can serve as a donor in ZnO lattice and increase the wide band gap which will improve the electrical and optical properties of ZnO. Al-doped ZnO is very useful in fabrication of optoelectronics devices and detectors [15,16]. Ozgur et al. has reported that the UV emission of un-doped ZnO NSs can be altered to blue emission by controlling the bandgap and oxidation states of Zn [17]. The blue peaks consisted of 3 subpeaks centered at 422, 441 and 470 nm. Kim et al. has reported that these peaks originated from Zn interstitials, vacancies and interface trap sites in ZnO NSs. Decreasing the particle size of Al-doped ZnO NSs has also produced shifted blue peaks from 420 to 441 nm [18]. Nevertheless decrease in green emission has been noticed in Al-doped ZnO. Increased concentration of Al-doped ZnO exhibited increased full-width at half-maximum (FWHM). Lupon et al. has reported that, this mainly due to direct transitions of electrons between the conduction band and valence band tails [19]. Bahedi et al. presented that Al-doped ZnO showed strong PL intensity in UV emission region compared to un-doped ZnO with samples that exhibited good crystallinity [20]. There have been reports on the effect of Al doping on the band gap shift of ZnO. Many have reported trends of increasing optical band gap with increasing Al doping levels which was mainly due to the Burstein-Moss effect [21]. Using uv-vis spectroscopy, Jun et al. [22] reported slight shift from 3.28 eV for pure ZnO to 3.29 eV for Al-doped ZnO at 2 at. % synthesized using the sol gel technique [21]. Greater shift value to 3.44 eV was reported by Khan et al. [23], at the same concentration, also using the sol gel synthesis technique. In another study on rf sputter coated Al-doped ZnO films, optical band gap varied from 3.57 to 3.67 eV for Al doping level of 2 to 4 at. % [24]. Also, they have reported a decrease in band gap values to 3.52 and 3.46 eV respectively for Al doping level of 2.5 and 3 at. %. However it increased to 3.6 eV at 3.5 at. % Al doping level.

Many techniques for the preparation of ZnO have been used including physical evaporization [25], chemical vapor deposition [26,27], solvothermal [28], carbothermal method [29] and flame synthesis [30,31] on different substrate materials. Most of the techniques involved mixture of pure ZnO powder and Al source solution in order to produce Al-doped ZnO. In late 90’s methanol was used as a source of oxygen and most of the studies have been reported at low temperatures on selected transition metals surfaces. Rufael *et al*. have reported that methanol undergoes thermal dissociation on iron, Fe (110) surface to form adsorbed methoxy (CH_3_O^-^) species and hydrogen at 100 K [32]. Levis et al. also have reported about thermal decomposition of methanol but on palladium, Pd (110) surface, methanol decomposed into CH_3_O_(a)_ species with an additional of CH_3(a)_ and H_2_O_(a)_ at 125 K [33]. Both Rufael and Levis have described that the CH_3_O_(a)_ species undergoes dehydrogenation to form CO and H_2_ which later leads to formation of ZnO NSs. Harikumar *et al*. have studied C-O bond scission of methanol, ethanol and 2-propanol on polycrystalline Zn metal surfaces. X-ray photoelectron spectroscopy (XPS) and vibrational electron energy loss spectroscopy (VEELS) were able to trace the co-adsorption of methanol and C-O bond scission of ethanol at relatively low temperature on the polycrystalline Zn surface which giving rise to a hydrocarbon species and oxygen [34].

In this work we used a variation of experimental method as reported earlier [35]. This technique involves the combination of VPT of methanol and thermal evaporation of CuZn to obtain large area deposition that consumes reduced time of reaction and would be feasible in growing doped and un-doped ZnO at suitable temperature on silicon (Si) substrate. Methanol was introduced as a source of oxygen whilst CuZn alloy as source of Zn and as the catalyst in obtaining doped and un-doped ZnO NSs [36]. Technically we can use any scrap alloy material that contain Zn. In addition functionalized ZnO NSs can be produced using the VPT technique that has been introduced on selective substrates.

## Experimental

**Fig 1:**
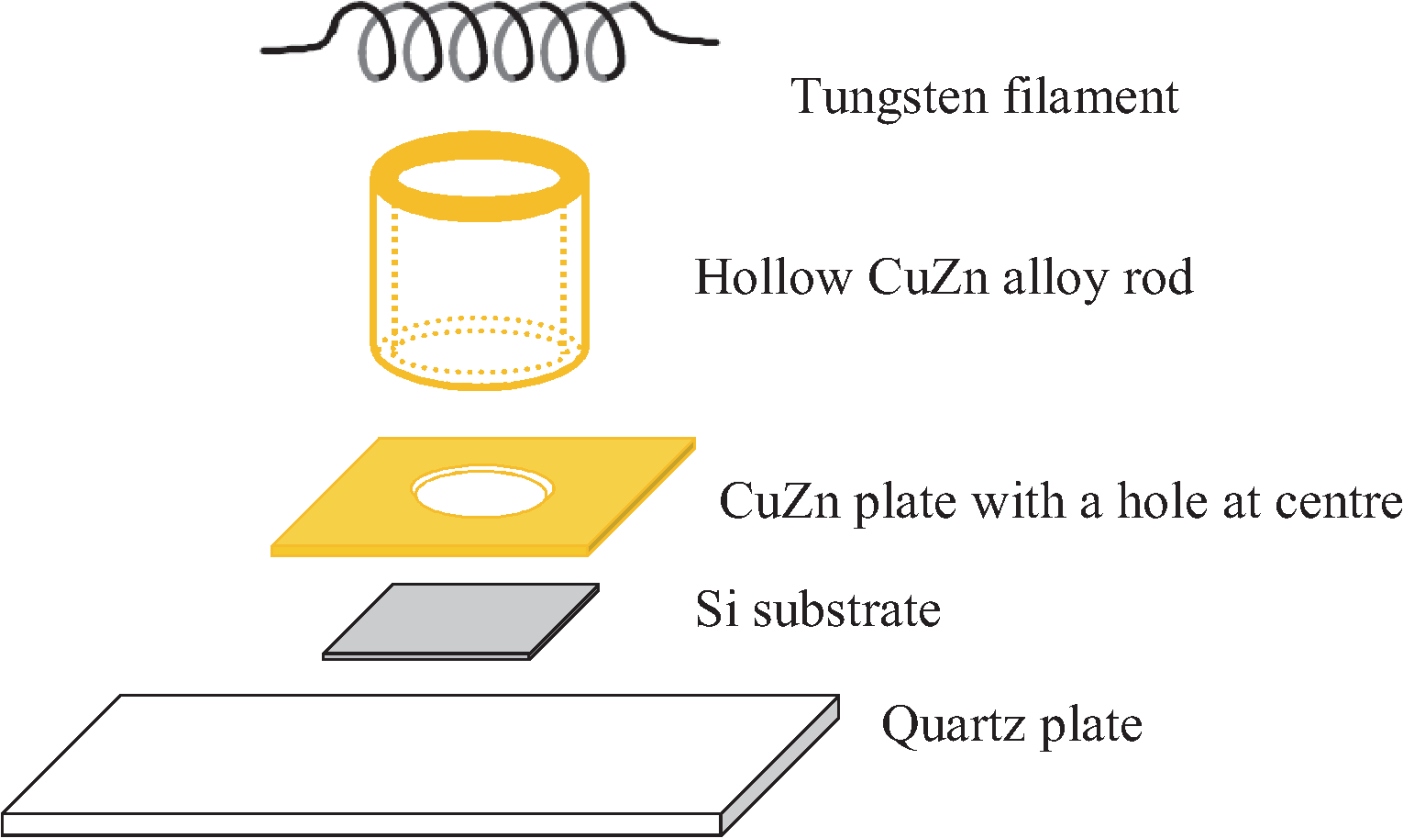
Illustration of experimental setup in order from bottom: quartz plate, Si substrate, CuZn plate, hollow CuZn alloy cylinder and tungsten hotwire in a vacuum chamber.

The un-doped and Al-doped ZnO NSs were grown on mirror finished Si (001) substrate. CuZn alloy was used as Zn source whereas methanol was used as source of oxygen in this work. The Si wafer was cut into chips about 1.5 by 1.5 cm. The oxide layer on the Si chip was removed using hydrofluoric (HF) acid solution. Then it was cleaned ultrasonically in acetone bath solution for about 30 minutes to remove any presence of HF stain and impurities on the surface. Then the Si chips were further rinsed in de-ionized (DI) water to remove any existing acetone stain and impurities on the chips. The Si chips were immediately let dried in a desiccator at room temperature before placed onto a cleaned quartz slide. A CuZn alloy plate thickness of 0.5 mm with a dimension of 2.3 by 2.3 cm and 0.5 cm diameter hole in middle was cleaned ultrasonically in acetone bath and rinsed with DI water before placing it on the Si chip. On top was this, a homemade two-sided hollow CuZn alloy rod was placed to cover the whole surface of the Si chip as per illustrated in Fig. 1.

Argon (Ar) was flowed at 100 sccm through mixture of methanol (50g) and acetone (4g) solution to allow methanol vapor is transported into the deposition chamber to produce un-doped ZnO NSs. Acetone was used in order to prevent deterioration of tungsten filament that would contaminate the samples. Al-doped ZnO NSs were obtained by adding desired aluminium nitrate (Al(NO_3_)_3_) into the mixture of methanol (50g) and acetone (4g) solution. Al(NO_3_)_3_ which has solubility rate 14.45g in 100 ml of methanol was used. The gas tubing systems were filled with drying agent, potassium silicates to allow dried methanol vapor and Ar into the deposition chamber. This methanol vapor underwent thermal dissociation under tungsten hotwire which is heated to 1800°C. Copper (Cu) in the CuZn did not underwent thermal evaporation due to its higher melting point compare to the operating temperature. Zn in CuZn was thermally evaporated at temperature greater than 395°C which then readily condensed on Si substrate. The temperature of Si was monitored at 800°C during the deposition period of 30 minutes. At the end of 30^th^ minute the methanol transportation was halted and the heated hotwire was switched off but continuous flow of Ar at 100 sccm without flowing through the mixture of methanol and acetone solution was maintained until the temperature of substrate drop to room temperature. The influence and possibility of Al doping in ZnO NSs were investigated by varying the Al weight (wt) percentage of 0.73, 1.28 and 1.82.

The samples were characterized using the FESEM Leica S440, XRD using Cu *Kα*, 1.54 Å radiations, photoluminescence (PL) and Raman scattering characterization. The PL study was carried out using Renishaw inVia Raman microscope at room temperature using a 325 nm helium-cadmium (He-Cd) laser light which passes through three visible lens sets and a diffraction grating of 1200 lines/mm whereas for Raman a green laser excitation wavelength of 532 nm was used.

## Results and Discussions

### FESEM

**Fig 2:**
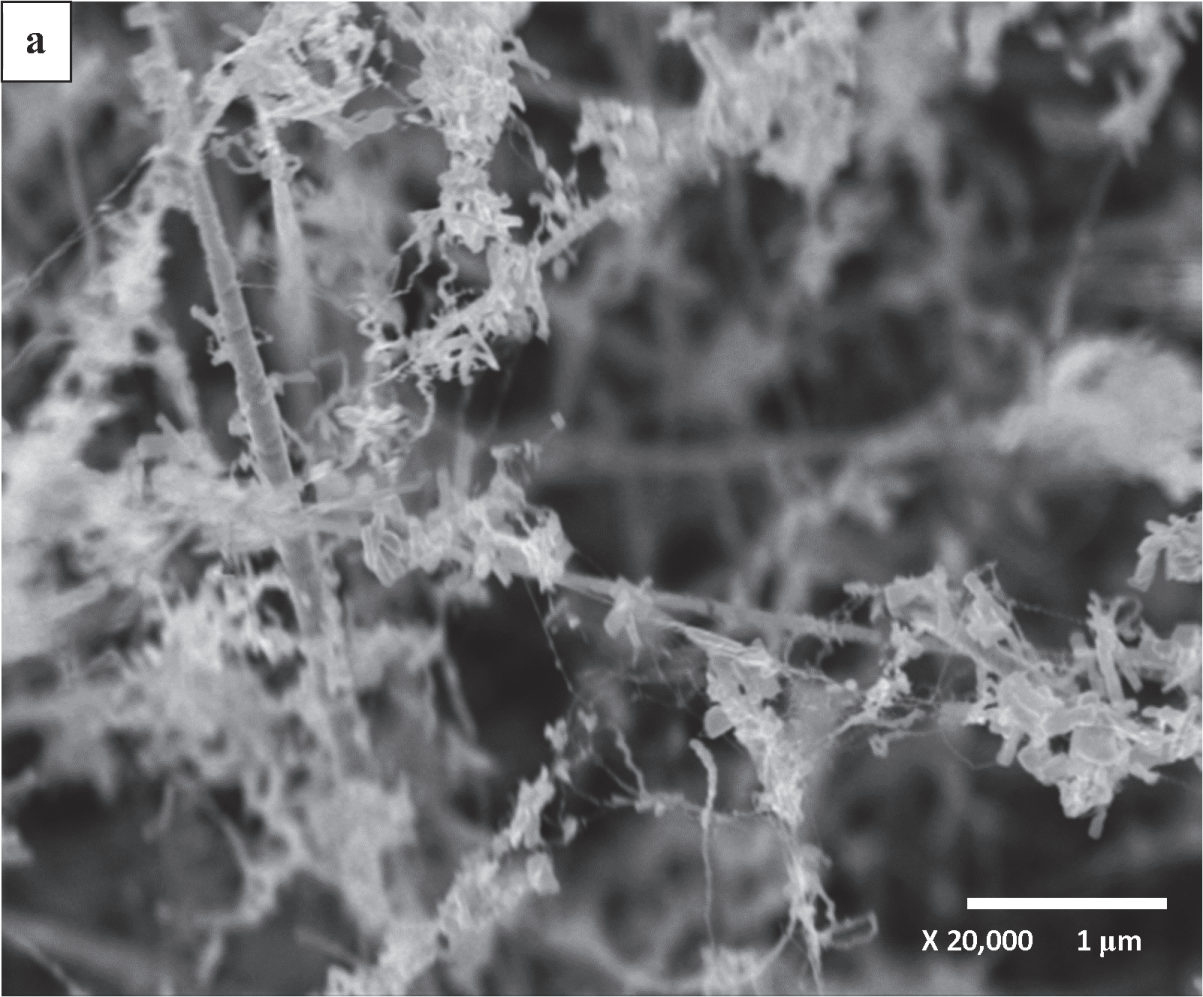
FESEM images of (a) un-doped ZnO NSs and the respective (b) X100K magnified image.

**Fig 3:**
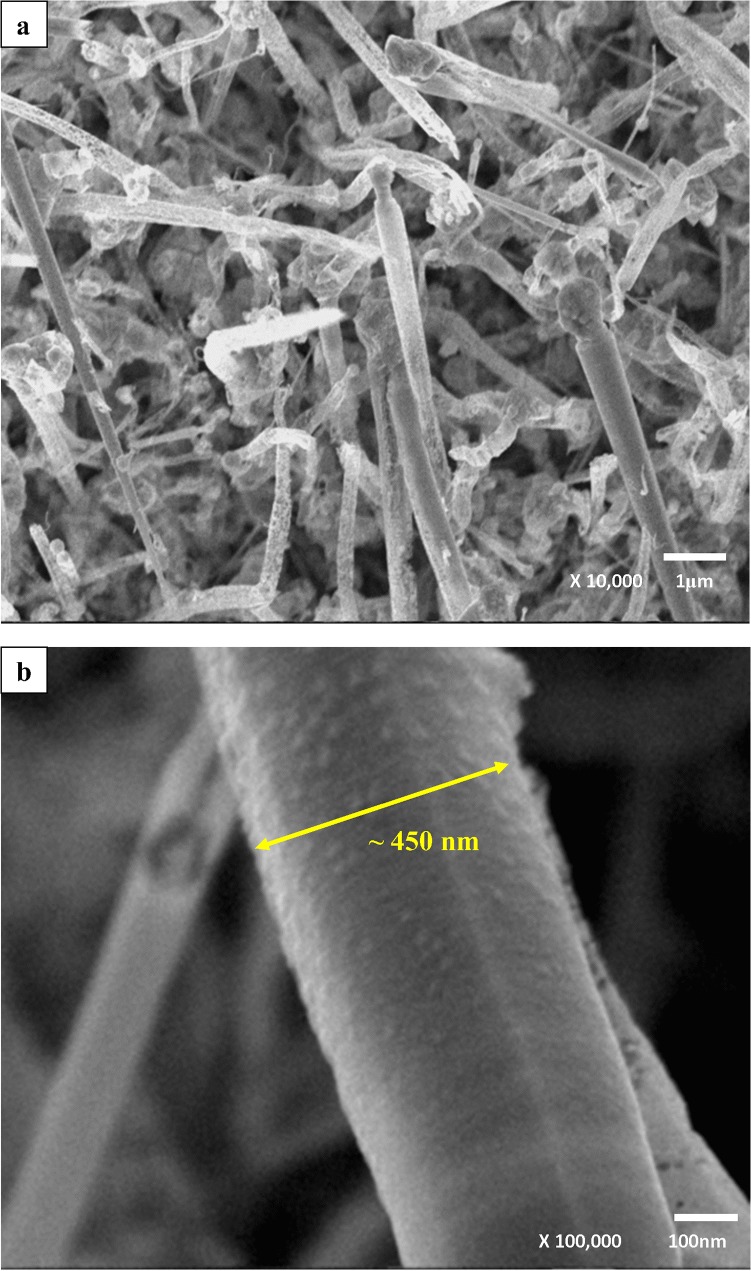
FESEM images of Al-doped ZnO NSs wt. % of (a) 0.73, (c) 1.28 and (e) 1.82. The respective X100K magnified images are (b), (d) and (f).

Fig. 2(A) shows the FESEM images for un-doped ZnO NSs. Presence of tiny ZnO nanostrings, nanoflakes and nanowires are observable in the image. The magnified version shown as Fig. 2(B) reveals the size of ZnO tip is about 10 nm. In our earlier report on the growth time dependence the un-doped ZnO NSs showed growth from button-mushroom at growth time of 5 and 10 minutes to nanoneedle structures as growth time increased to 30 minutes [35]. However significant changes were identified in Al-doped ZnO NSs grown for 30 minutes at Al wt. % of 0.73, 1.28 and 1.82 as shown in Fig. 3 (A, C and E). The Al-doped ZnO NSs revealed growth like button-mushroom compared to nanoneedle structure in the un-doped ZnO NSs. In addition uncommon features in the images of Al-doped samples are in comparable with the un-doped ZnO NSs. The effect of Al incorporation is noticeable on the wall of ZnO as in Fig. 3(B, D and F). The magnified images of Al-doped ZnO NSs shows diameter decreasing trend 450, 400 and 300 nm with an increasing Al wt. % of 0.73, 1.28 and 1.82. These diameters values are almost 4 times greater than that of un-doped ZnO NSs. Fig. 3(B) depicts formation of tiny bumps on the wall of ZnO whereas Fig. 3(D) shows few rows of grooves and protuberance grown on the stem of the button-mushroom like Al-doped ZnO NSs. The effects of Al on the wall of ZnO NSs found to be obvious as the Al wt. % was increased to 1.82 as in Fig. 3(F). This could be due to doping effect in ZnO NSs.

### XRD

**Fig 4:**
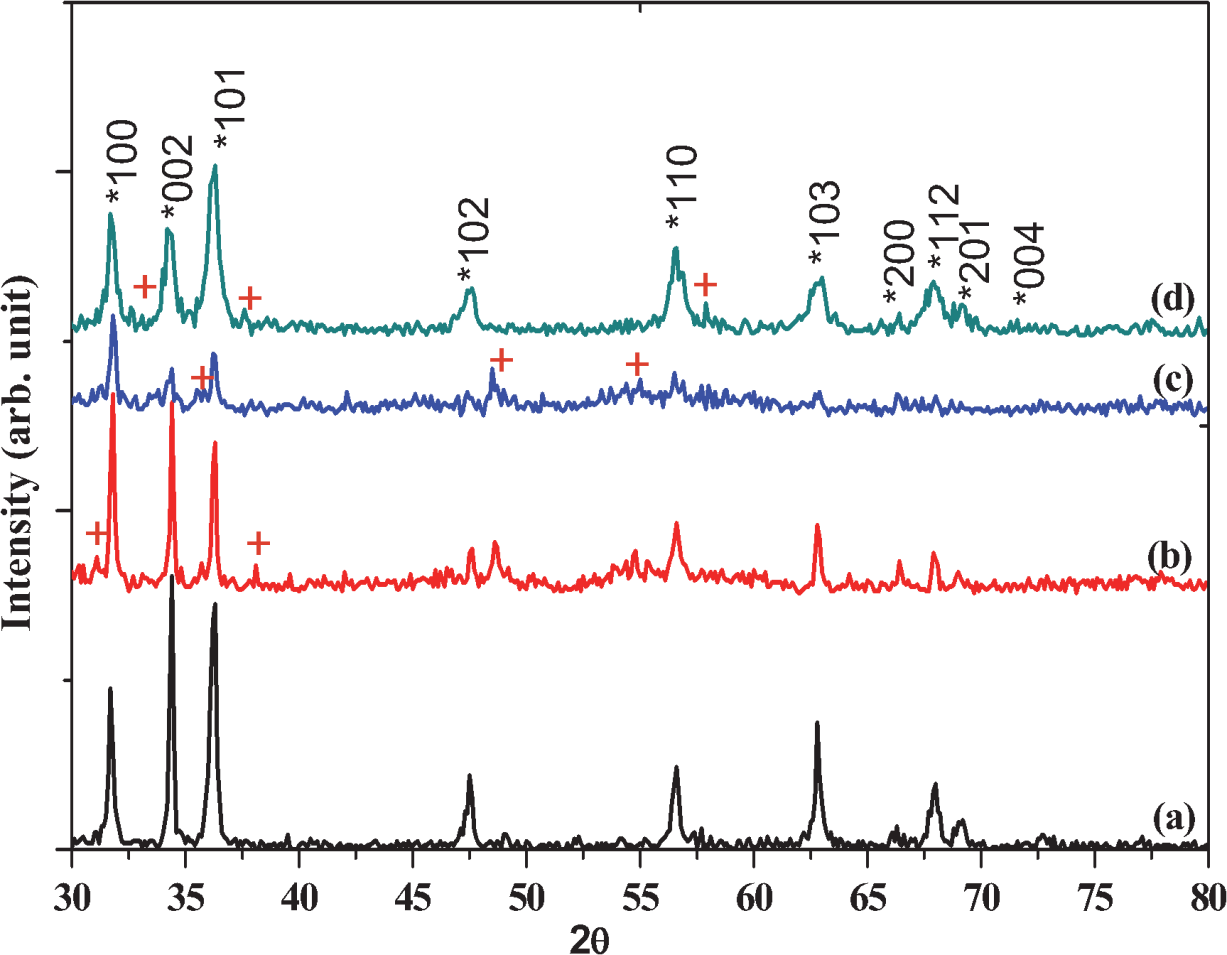
XRD spectra of un-doped ZnO and Al-doped ZnO NSs with Al concentration (b) 0.73, (c) 1.28 and (d) 1.82 wt. %.

The XRD spectra of Al-doped ZnO NSs prepared at various Al concentrations 0.73, 1.28 and 1.82 wt. % were compared with the un-doped ZnO NSs as in Fig. 4. All the detectable peaks can be indexed as hexagonal wurtzite ZnO that found as in the standard reference data JCP2.2CA: 00-036-1451. The XRD profile strongly shows various crystalline natures of Al-doped ZnO NSs compared to the un-doped ZnO NSs. There are additional peaks observable in the XRD spectra of Al-doped ZnO NSs which are marked as “**+”**in Fig. 4. These peaks can be attributed incorporation of Al^3+^ of ionic radius 53 pm which is smaller than the Zn^2+^ (74 pm) is successfully incorporated into ZnO NSs. However no other impurities detected except peak splitting which is noticeable in the XRD profile.

Significant changes in the intensity and FWHM were noticed from the three dominant peaks (100), (002) and (101) which could be due to the effect of Al doping into the nanostructures of ZnO NSs [37]. Preferential growth direction changes as the doping concentration of Al was increased. The Al-doped ZnO NSs of 0.73 wt. % shows the highest (002) diffraction peak intensity. More preferential growth direction at <101> is noticeable at higher Al concentration of 1.82 wt. % compared to the un-doped ZnO which shows preferential growth direction in <002>. The presence of Al changed the diffusion rate of Zn and O on the Si substrate during deposition. Thus, it alters the energetic balance between (002) and (101) orientations which further lead to weakening of (002) orientation and preferred (101) texture orientation appeared [38]. The peak intensities of Al-doped ZnO of 0.73 and 1.28 wt. % found to be lower compared to the un-doped ZnO NSs. This is indicating that Al atom has occupied the position of Zn in the ZnO lattice as a substitutional atom. The effect of Al doping also can be observed as a minute variation in the 2θ values of diffraction peaks of (100), (002) and (101). Table 1 shows detailed Gaussian deconvoluted peaks for the Al-doped ZnO NSs which were compared with the un-doped ZnO. Peak center of ZnO:Al 0.73 wt. % found to be shifted to the right relative to the un-doped ZnO NSs. The Al-doped ZnO of 1.28 wt. % shows right shift for (100) and (101) whereas (002) found to be shifted to left. Further increased of Al concentration to 1.82 wt. % resulted right shift for (100) and left shift for (002) and (101).

**Table 1:** Details of Gaussian deconvoluted peaks of un-doped and Al-doped ZnO.

ZnO Diffraction plane	XRD Parameters	ZnO Sample (Al wt. %)			
		Un-doped (0.00)	Al-doped (0.73)	Al-doped (1.28)	Al-doped (1.82)
**(100)**	Peak center (±0.001°)	31.716°	31.780°	31.791°	31.728°
	Peak shift ***(Δ 2θ)***	***0*.*00***	***+0*.*064°***	***+0*.*075°***	***+0*.*012°***
	FWHM	0.207°	0.169°	0.208°	0.466°
**(002)**	Peak center (±0.001°)	34.387°	34.405°	34.337°	34.320°
	Peak shift ***(Δ 2θ)***	***0*.*00***	***+0*.*018°***	-***0*.*050°***	-***0*.*067°***
	FWHM	0.189°	0.106°	0.298°	0.373°
**(101)**	Peak center (±0.001°)	36.227°	36.241°	36.249°	36.188°
	Peak shift ***(Δ 2θ)***	***0*.*00***	***+0*.*014°***	***+0*.*022°***	-***0*.*039°***
	FWHM	0.316°	0.134°	0.213°	0.518°

The peaks shift toward high angle as compared to the standard ZnO XRD pattern, indicating the replacement of the larger Zn^2+^ (74pm) by the smaller Al^3+^ (53pm) in the ZnO lattice and the formation of the smaller Al-doped ZnO lattice [39,40]. Higher deviation of (100) diffraction peak in Al-doped ZnO wt. % of 0.73compared to 1.28 and 1.82 wt. % attributes high concentration of defects inside the Al-doped ZnO lattice [41].

Peak broadening and noise in the Al-doped ZnO NSs were obvious for the increased Al concentration. Although high intensity and narrower peak broadening often related to high crystal quality [42] as in Al-doped ZnO 0.73 wt. %, identification of noise and peak shift shows that deterioration of crystal quality in all Al-doped ZnO NSs samples. Greater peak broadening is noticeable in Al-doped ZnO NSs 1.82 wt. %. This reflects non-uniform strain due to incorporation of Al atom into lattice of ZnO which can be attributed to the deterioration of crystallinity of Al-doped ZnO NSs [43]. Therefore, the deterioration of crystallinity is the evidence for the incorporation of Al^3+^ replacing the greater radii Zn^2+^ into the ZnO NSs lattice.

### Photoluminescence

**Fig 5:**
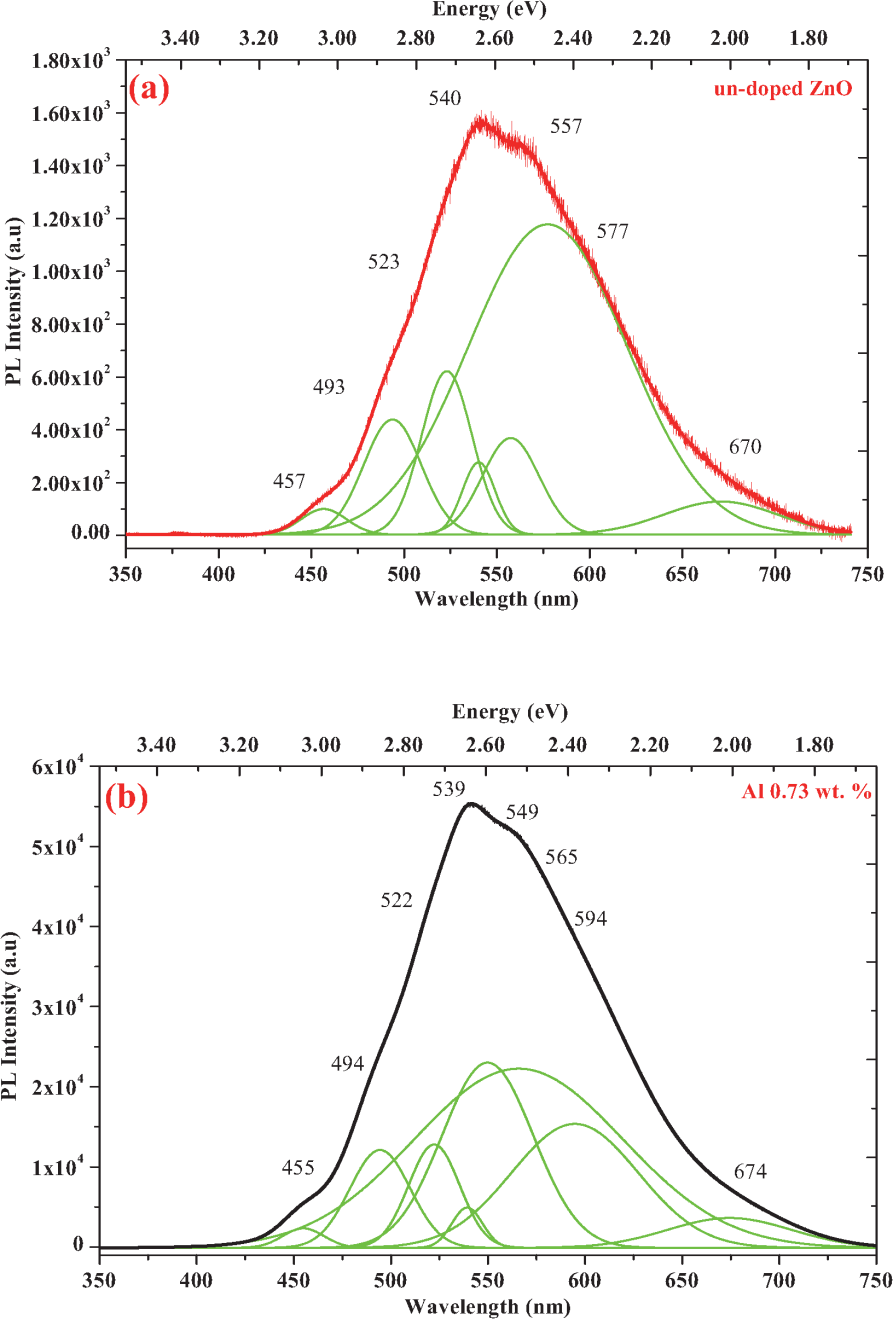
The Gaussian curve fitted green luminescence PL spectra of (a) un-doped ZnO and Al-doped ZnO wt. % of (b) 0.73, (c) 1.28 and (d) 1.82.

Fig. 5 shows the PL profile for un-doped and Al-doped ZnO NSs of 0.78, 1.28 and 1.82 wt. % at room temperature. The effect of Al doping into the ZnO NSs shows significant changes at NBE, DL and NIR emission regions. This visible PL emission in the un-doped ZnO NSs can be attributed to different intrinsic defects such as Zn_i_, O_i_, V_zn_, V_Zn_^-^, V_o_, V_o_^+^, O_Zn_ [44]. Nevertheless, defects due to carbon incorporation in the un-doped ZnO NSs were also responsible as reported earlier by Chrissanthopoulos et al. [45]. The PL profile attributes broad peak at range from 430 to 750 nm which is subjected to dominant green luminescence (GL) band. GL band centered at around 540 (2.28 eV) observed for all Al-doped and un-doped ZnO NSs was due to the radial recombination of photo-generated hole with the electron of singly ionized charged particles in the V_o_ [46,47]. All the Al-doped ZnO NSs samples show higher intensity compared to the un-doped ZnO NSs. However, reduction in the PL intensity of GL band was observed as the Al concentration increased [19]. This is due to the evidence of O and Zn defects in the NSs. Although many works had been reported, some have proved that V_o_ is an unlikely contributor to GL band luminescence compared to V_zn_^2-^ based on the electron transition state [48, 49]. V_zn_^2-^ is the most energetically favorable and stable defects to be formed in the ZnO NSs. A similar outcome has been reported by Wang et al. for ZnO thin film sample annealed at 850°C [43].

A small broad shoulder around 557 nm (2.22 eV) in the un-doped ZnO, which can be attributed to yellowish-green (YG) band observed at 565 and 568 nm in Al-doped ZnO of 0.73 and 1.28 wt. %. The YG band shoulder found to be broader with the increased wt. % of Al. Whereas in Al-doped ZnO of 1.82 wt. %, the YG band found to be broader with a peak centered at 548 nm (2.26 eV). A closer look at the deconvoluted PL profile as in Fig. 5(D) exhibited most intense peak at 596 nm (2.09 eV) that relates to yellow luminescence (YL) near to orange region. This is due to the substitution of Zn^2+^ (74 pm) with smaller radii Al^3+^ (53 pm) in the NSs of ZnO. Besides that, the intensity ratio of green to YG emission found to be increased as the wt. % of Al increased. This significantly shows the incorporation of Al^3+^ into the texture of ZnO NSs. Recombination of donors with Al acceptors in the lattice of ZnO NSs seem to be the cause of YG luminescence.

**Fig 6:**
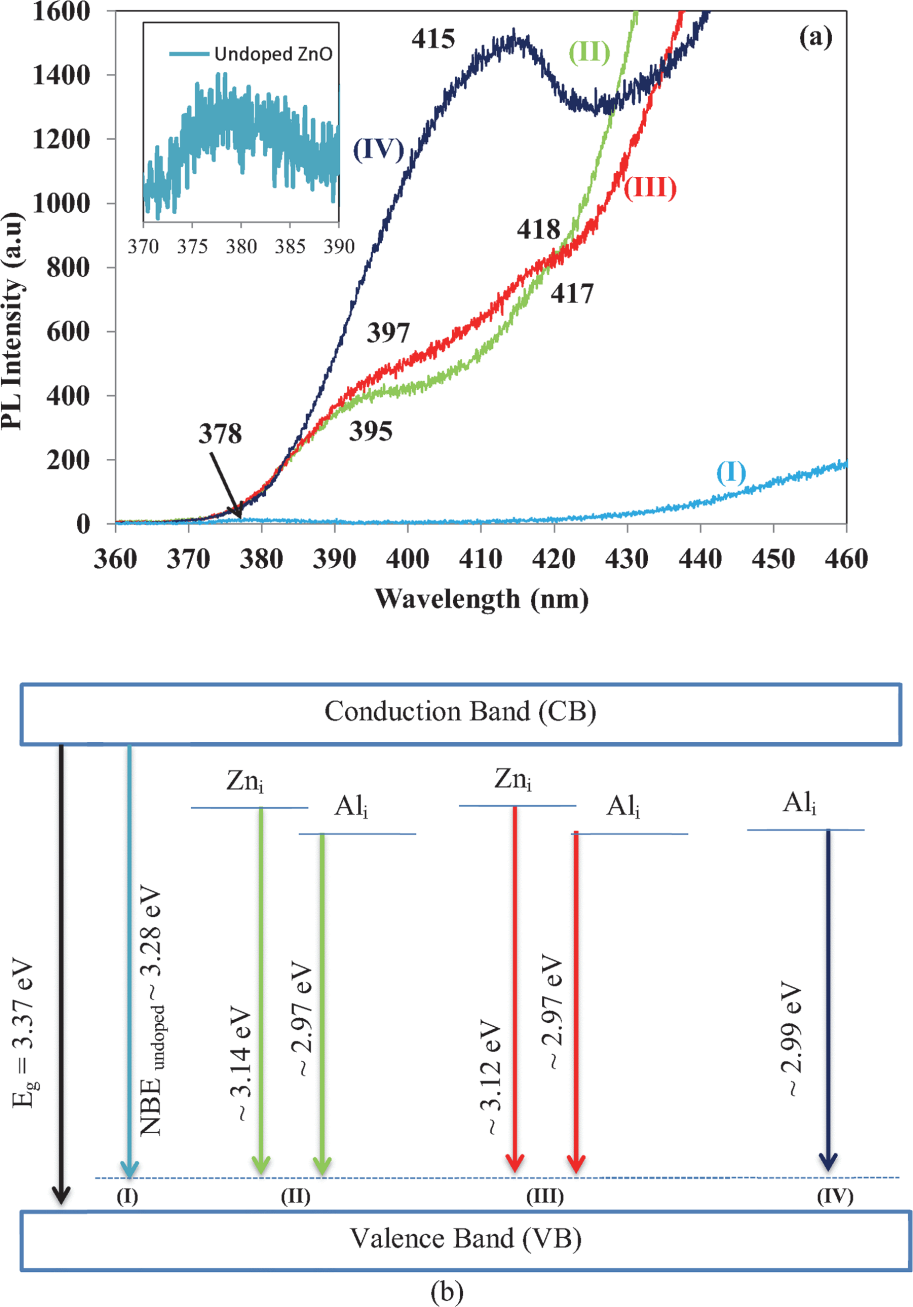
(a) NBE PL spectra and (b) Schematic band diagram of NBE based on PL data of (I) un-doped ZnO and Al-doped ZnO wt. % of (II) 0.73, (III) 1.28 and (IV) 1.82 at NBE.

Significant changes were identified at NBE range 360–430 nm which corresponds to the recombination of free excitons through exciton-exciton collision process. The magnified x-axis in range 370–390 nm for the un-doped ZnO NSs is shown as inserted spectrum in Fig. 6(A).The un-doped ZnO NSs exhibits UV emission band ∼378 nm (3.28 eV) in the inserted spectrum. Broad shoulder ∼395 and ∼417 nm for Al-doped ZnO 0.73 wt. % whereas 397 and 418 nm for Al-doped ZnO wt. % of 1.28 attributes UV emission and violet luminescence (VL) region respectively. The UV is red shifted from 378 nm to 395 and 397 nm in Al-doped ZnO is due to Zn_i_ [50]. The VL and noticed to be sharper ∼415nm (2.99 eV) as the Al wt. % increased to 1.82. It is clear that the NBE was violet shifted due to Al doping. The combined effect of optical transition to the excitonic state of ZnO and electronic transitions involving crystal-field split is responsible for the violet shift. The shift of UV to VL can be ascribed to Al_i_ beside the Zn_i_. This was generated by the substitution of Zn sites with Al atoms [51]. Shan et al. have reported similar VL band in Al-doped ZnO prepared at room temperature compared to Al-doped ZnO annealed at 500°C which attributes UV emission at 377 nm [52]. The schematic band diagram of the NBE emission is shown in Fig. 6(B).

**Fig 7:**
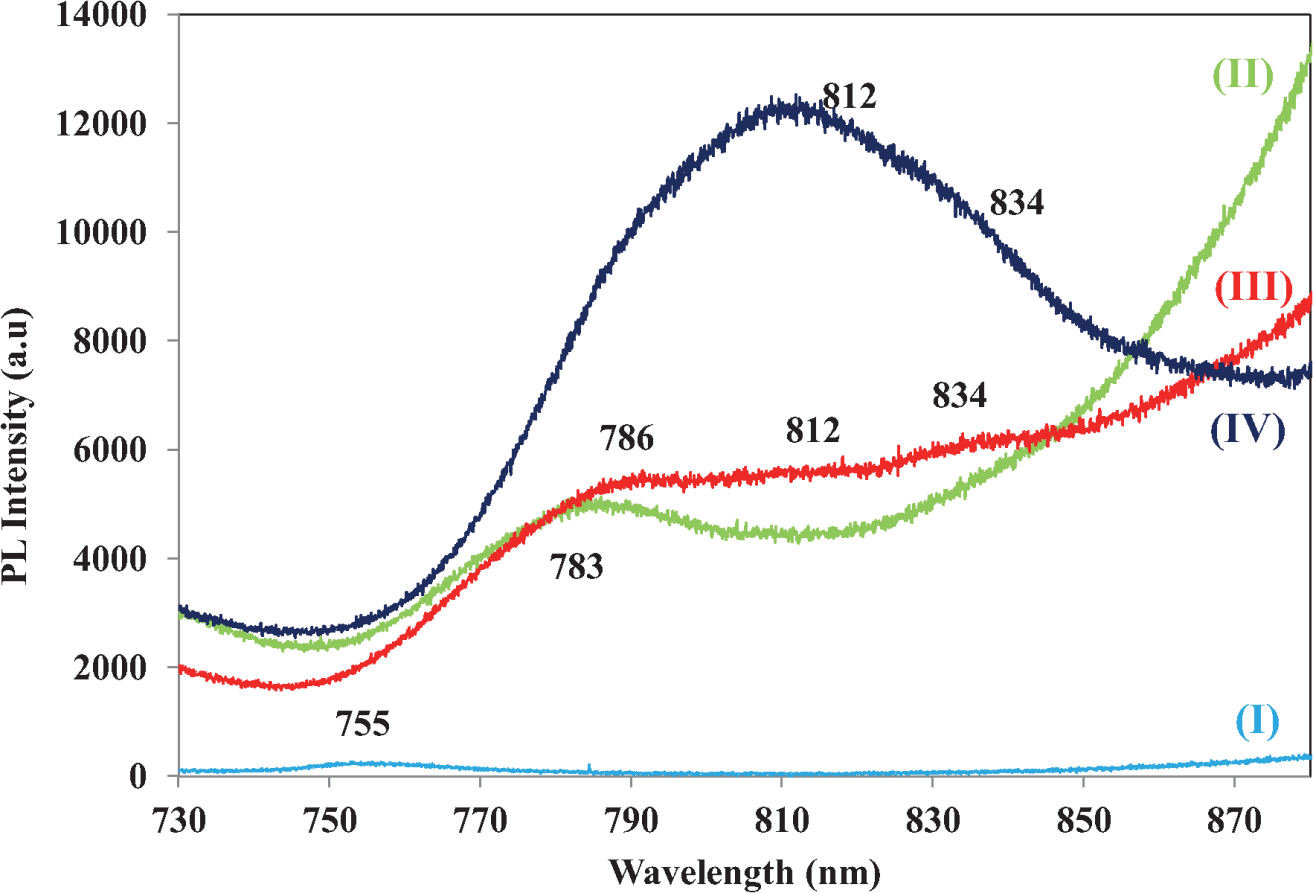
PL spectra of (I) un-doped ZnO and Al-doped ZnO wt. % of (II) 0.73, (III) 1.28 and (IV) 1.82 at NIR.

Red luminescence at 755 nm (1.64 eV) in un-doped ZnO NSs observed to be far to the left which is blue shifted compared to the Al-doped ZnO NSs that shows NIR emissions as in Fig. 7. The Al-doped ZnO NSs of 0.73 wt. % shows small broad shoulder ranged from 750–800 nm with a peak centered at 783 nm (1.58 eV). The 1.28 wt. % of Al-doped ZnO shows broader shoulder ranged from 750–850 nm compared to the Al-doped ZnO 0.73 wt. %. Three bulging peaks were identified at 786, 812 and 834 nm in Al-doped ZnO 1.28 wt. %. This shows transition of free excitons occurred in ZnO NSs doped with Al wt. % of 1.28. Significant change is observable in the PL profile of Al-doped ZnO 1.82 wt. %. Dominant NIR band from 750–880 nm with a peak centered at 812 nm (1.53 eV) could be due to incorporation of smaller radii Al particle into lattices of ZnO.

### Raman scattering

**Fig 8:**
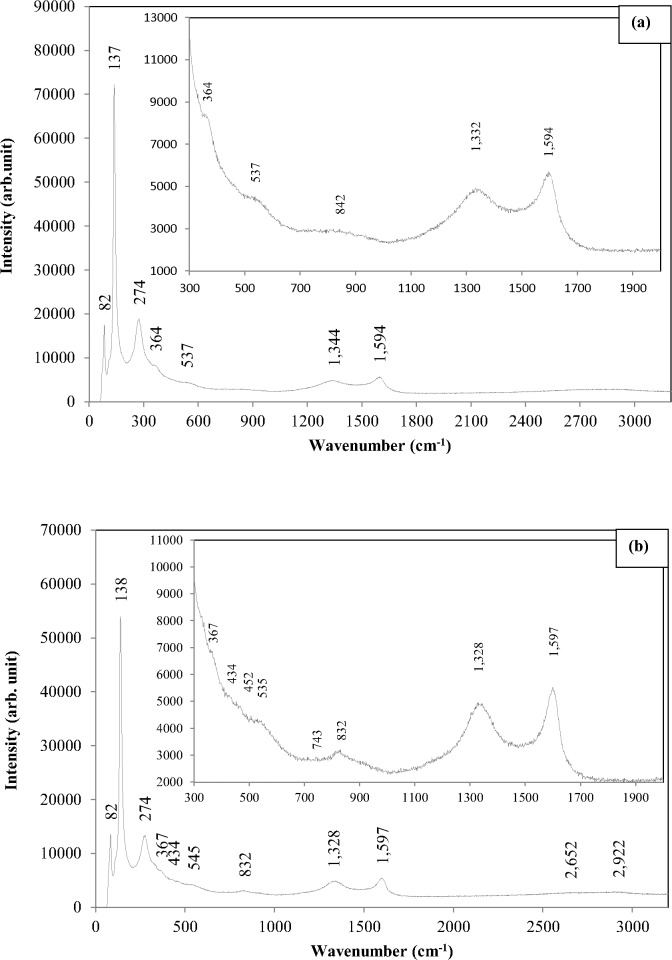
Raman scattering of Al-doped ZnO NSs with Al concentration (a) 0.73, (b) 1.28, (c) 1.82 wt. % and (d) un-doped ZnO NSs.

The Raman scattering result of un-doped ZnO NSs which was discussed earlier [53] is compared to Al-doped ZnO NSs. The Raman scattering of Al-doped ZnO of 0.73, 1.28 and 1.82 wt. % and un-doped ZnO NSs that were prepared for 30 minutes of deposition period are shown in Fig. 8. The Raman scattering profile exhibits significant changes in the peak positions as compared between Al-doped and the un-doped ZnO NSs samples. Generally the Raman spectral intensity of the Al doped ZnO NSs for 0.73, 1.28 and 1.82 wt. % were profoundly decreased in all the measured frequency regions compared to that of the un-doped ZnO NSs as has been reported elsewhere [54,55].

Peaks that can be related due to the vibration of Zn sublattices (below 300 cm^-1^) were found at around 82, 109, 138 and 274 cm^-1^ in all Al-doped ZnO samples. Raman peaks around 82 and 109 cm^-1^ found to be similar to the un-doped ZnO but the peak at 109 cm^-1^ observed to be diminished with the increased Al concentration compared to the un-doped ZnO. These two peaks in un-doped ZnO, 82 and 109 cm^-1^ can be attributed respectively to E_2_ mode and incorporation of C clusters from methanol solution. This finding is compatible to a work reported by Khanderi et al. [53] which involved fabrication of ZnO NSs decorated with multi wall carbon nanotubes (MWCNT).

The dominant sharp peaks 145 cm^-1^ in un-doped ZnO which is detectable in the room temperatures Raman scattering are contradictory to Cusco et al. [56]. They also observed the same peak and have reported that intensity enhancement and sharpening of the peak at 142 cm^-1^ as Raman scattering was done at lower temperatures at 80 K due to local mode origin of the peak. This peak is noticed to be shifted to an anomalous mode around 138 cm^-1^ in Al-doped ZnO of 0.73, 1.28 and 1.82 wt. %. This anomalous mode can be attributed to the effect of Al doping in ZnO NSs. In the un-doped ZnO, the A_1_ symmetry peak 288 cm^-1^ that can be assigned to B_1_^high^-B_1_^low^ mode also found to be shifted to another anomalous mode at peak 274 cm^-1^. These two anomalous peaks 138 and 274 cm^-1^ which were shifted to low wavenumber can be related to intrinsic host lattice defects in ZnO where the defect has become activated vibrating complexes due to the incorporation of Al dopants [57]. The shift also can be regarded as stress or strain induced by Al ion in the ZnO lattice.

The number of frequencies related to O modes in the range of 300–700 cm^-1^ found to be much less pronounced in the Al-doped ZnO NSs compared to the un-doped ZnO NSs. The E_2_^high^ mode for the Al-doped ZnO NSs of 1.28 and 1.82 wt. % was shifted to 434 and 433 cm^-1^ whereas the un-doped ZnO NSs shows profound peak at 439 cm^-1^. But the E_2_^high^ mode is not detectable in ZnO:Al 0.73 wt. % as in Fig. 8(A). The A_1_ symmetrical peak 340 cm^-1^ in the un-doped ZnO NSs attributes a good agreement with E_2_^high^—E_2_^low^ frequencies. A great shift about 26 cm^-1^ was noticed in the ZnO:Al NSs which are to 364, 367 and 366 cm^-1^ for the respective Al concentration of 0.73, 1.28 and 1.82 wt. %. Peak 578 cm^-1^ can be revealed to resonance Raman scattering whereas which is close to A_1_(LO) mode of ZnO quantum dots [58]. This peak in the proximity of A_1_(LO) were assigned to surface optical modes predicted theoretically and O vacancies, Zn_i_ or combination of the two [59].

The Al-doped ZnO NSs exhibited small broader shoulder at around 535 cm^-1^ whereas in the un-doped ZnO NSs it found to be diminished. They can be assigned to A_1_ symmetry and attributed to 2B_1_^low^ and LA overtones along L, M and H lines. Presence of Al has bulged out to be broad shoulder. The undetectable E_1_-LO mode in the un-doped ZnO NSs at 590 cm^-1^ was only detected in Al-doped ZnO of 1.82 wt. % compared to Al-doped ZnO of 0.73 and 1.28 wt. %. This could be due to Al incorporation into ZnO has triggered the vibration frequency of O related sublattices in ZnO. An anomalous mode which was detected in the un-doped ZnO at 832 cm^-1^ turn out to be more profound in Al-doped ZnO NSs of 1.28 wt. % compared to Al-doped ZnO NSs of 0.73 and 1.82 wt. %. This mode could be due to the influence of Al and the C clusters from methanol. Other peaks that have been detected and discussed earlier in un-doped ZnO NSs in section 5.4.4 such as 866, 923, 941, 962 and 986 cm^-1^ noticed to be vanished in all Al-doped ZnO NSs. Peak at 1090 and 1051 that can be assigned to be TO+LO combination modes respectively at M, L and A, H points were vanished in Al-doped ZnO. However the 1051 cm^-1^ could be shifted to 1061 cm^-1^ as detectable only in the Al-doped ZnO NSs of 1.82 wt. %.

The Raman absorption for the disordered D and G band which is detectable respectively at 1337 and 1575 cm^-1^ in the un-doped ZnO NSs can be related to presence of C clusters from methanol solution that has decorated the wall of ZnO NSs. But intensity enhancement was noticed in all Al-doped ZnO NSs. The disordered D band was identified as 1332, 1328 and 1334 cm^-1^ whereas the disordered G band was 1594, 1597 and 1596 cm^-1^ for Al-doped ZnO of 0.73, 1.28 and 1.82 wt. %. The intensity ratios of D band (I_D_) to G band (I_G_), (I_D_/I_G_) were calculated to be 0.86, 0.90 and 0.89. The low value of I_D_/I_G_ shows low disordered degree in the low concentrated Al-doped ZnO of 0.73 wt. %. Increasing the Al concentration in methanol highly has influenced the incorporation of C clusters in decorating the wall of ZnO NSs.

### Proposed growth mechanism

The growth mechanism which involves a series of chemical reaction is proposed based on various outcomes that have been reported earlier by our group [30, 33] and elsewhere [60]. The thermally heated CuZn alloy up to temperatures 800°C in the Ar environment has allowed only Zn atoms to escape as Zn vapors at temperatures greater than 395°C. The presence of Ar environment at 100 sccm in the isolated system thermodynamically has rearranged and reduced the crystallite size of hexagonal ZnO [30,44].The Zn vapors which then condensed as Zn solid on Si substrate reacted with methanol vapors to form ZnO NSs.

$$\text{CuZn}\;\left(\text{solid brass}\right)\;\overset{>395{}^{\circ}\text{C}\;}{\to }\;\text{Zn}\;(\text{vapor})\;\overset{\;}{\to }\;\text{Zn}\;\text{film}$$

Al(NO_3_)_3_ in methanol, dissolves completely to form aluminium methoxide ((CH_3_O)_3_Al) which further dissolves in excess methanol. The enhancement of intensity for D and G band in Raman scattering proved that Al was chemically bonded to methanol. Here, the methanol also acts as oxidizer for Al. The growth of Al-doped ZnO NSs as were obtained by allowing (CH_3_O)_3_Al vapor as Ar was flowed at 100 sccm through the mixture of (CH_3_O)_3_Al solution via the two sided hollow CuZn. We believed that C, H and O atoms in (CH_3_O)_3_Al underwent a dehydrogenation process in the presence of Cu (in CuZn alloy) at 300°C [61–63].

$${\left({\text{CH}}_{3}\text{O}\right)}_{3}\text{Al}\;(\text{a})\;\overset{\text{Cu},\;300{}^{\circ}\text{C}\;}{\;\to }\;{\text{CH}}_{3}\text{O}\;(\text{a})\;+\;\text{Al}\left(\text{a}\right)$$

$${\text{CH}}_{3}\text{O}\;\left(\text{a}\right)\;\overset{\text{Cu},\;300{}^{\circ}\text{C}\;}{\;\to }\;\text{HCHO}\left(\text{a}\right)\;+\;\text{H}\left(\text{a}\right)$$

Based on that, Cu in CuZn has acted as an catalyst and oxidized the methanol vapor into an intermediate absorbable methoxy species (CH_3_O_(a)_) [64] and Al species (Al_(a)_). The CH_3_O_(a)_ is thermodynamically unstable and were readily converted to formaldehyde (HCHO) by releasing a H_(a)_. The higher bond energy of C = O in HCHO is thermodynamically unfavorable and the polarity of carbonyl group and its high basicity lowered the transition state energy of activation and therefore results in faster rate of reaction with readily presence species [65]. So the chemically unstable HCHO vapor easily reacts with Zn vapor or Zn atoms to that which have been thermally evaporated at temperatures above 395°C to form hexagonal ZnO NWs. Besides that, further oxidation of HCHO to HCOOH is not predicted due to O_2_ absence environment. The Al(a) condensed as dopant into the lattices of ZnO NSs as it dropped in Si substrate. Apart of this, C-O bond in CH_3_O_(a)_ was readily cleaved by Zn vapors [66]. Co-adsorption of O_(a)_ and Zn_(a)_ appeared to give rise to a reactive oxygen ions (O^2-^) and Zn ions (Zn^2+^) on the Si substrate. Thus Zn^2+^ ionically bond with O^2-^ to form ZnO NSs.

$${\text{CH}}_{3}\text{O}\;\left(\text{a}\right)\;\overset{\;}{\to }\;\text{O}\left(\text{a}\right)\;+\;{\text{CH}}_{3}\left(\text{a}\right)$$

$$\text{O}\left(\text{a}\right)\;+\;2{\text{e}}^{-}\;\overset{\;}{\to }\;{\text{O}}^{2-}$$

$${\text{Zn}}^{2+}\;+\;{\text{O}}^{2-}\;\overset{\;}{\to }\;\text{ZnO}$$

## Conclusion

Al-doped and un-doped ZnO NSs were successfully prepared on Si substrate using the VPT of mixture of methanol and acetone via thermal evaporation of Zn from two-sided hollow CuZn alloy. Based on the FESEM the Al-doped ZnO NSs at Al concentration of 0.73, 1.28 and 1.82 wt. % exhibited button mushroom like shape compared to the un-doped ZnO which exhibited nanoneedle like growth. The profound changes on the stem of Al-doped ZnO which was 4 times that of un-doped ZnO further proved effect and incorporation of Al^3+^ into the NSs of ZnO. Peak shifts and widening in FWHM of (100), (002) and (101) reflection planes in Al-doped ZnO were indications of non-uniform strain during growth which resulted from the incorporation of smaller ionic radii of Al^3+^ into the lattices ZnO NSs. The PL significantly attributed greater peak shift at NBE from 378 cm^-1^ in undoped ZnO to 415 cm^-1^ in Al-doped ZnO at 1.82 wt. % and the red luminescence peak shift from 755 cm^-1^ in undoped ZnO to 812 cm^-1^ in Al-doped ZnO at NIR emissions can be related to the evidence obtained from FESEM and XRD results. Presence of dominant 145 cm^-1^ in the un-doped ZnO confirmed decoration with C clusters on wall of ZnO. Besides that the enhanced D and G band in all Al-doped ZnO NSs shows possible functionalization and doping process has occurred in ZnO NSs. The shift of dominant Raman signal from 145 to 138 cm^-1^ in Al-doped ZnO NSs and presence of Raman signal at 743 cm^-1^ were the main features of Al^3+^ incorporation in the lattices of ZnO NSs. Additional Raman signal at around 274 cm^-1^ compared to the un-doped ZnO NSs has confirmed incorporation of Al^3+^ into the lattices of ZnO.
