# Correction: Enhanced Photoluminescence and Raman Properties of Al-Doped ZnO Nanostructures Prepared Using Thermal Chemical Vapor Deposition of Methanol Assisted with Heated Brass

**DOI:** 10.1371/journal.pone.0126189

**Published:** 2015-05-07

**Authors:** The PLOS ONE Staff

The following information is missing from the Funding section: This work was supported by the Ministry of Higher Education of Malaysia under the HIR (H21001-F00033), UM.C/HIR/MOHE/SC/33 and UMRG grant (RP008-13AFR).

The complete, correct funding information is: This work is supported by Peruntukan Penyelidikan Pascasiswazah (PPP) of University Malaya Grant No.: PS212/2009A and UMRG Grant No.: RG247-12AFR, as well as by the Ministry of Higher Education of Malaysia under the HIR (H21001-F00033), UM.C/HIR/MOHE/SC/33 and UMRG grant (RP008-13AFR). The funders had no role in study design, data collection and analysis, decision to publish, or preparation of the manuscript.

The publisher apologizes for this error.
